# Sonographic patterns of Peyronie's disease in patients with absence of palpable plaques

**DOI:** 10.1590/S1677-5538.IBJU.2017.0298

**Published:** 2018

**Authors:** Lucio Dell'Atti, Andrea Benedetto Galosi

**Affiliations:** 1 Department of Urology, Polytechnic University of Marche Region, University Hospital “Ospedali Riuniti”, Ancona, Italy

**Keywords:** Erectile Dysfunction, Ultrasonography, Penis

## Abstract

**Purpose:**

Non-palpable isolated septal plaques of the penis are likely present in a significant number of patients affected by erectile dysfunction (ED) and penile pain without deformity or curvature. The aim of this study was to evaluate the ultrasound (US) patterns observed in patients investigated for ED or penile pain without curvature.

**Materials and Methods:**

We reviewed the medical records of 386 patients who underwent an initial colour-Doppler ultrasonography (CDU) of the penis for DE and/or penile pain without curvature. After satisfying inclusion criteria, 41 patients were individualized. All patients had a non-palpable plaque with involvement of the penile septum. Three US patterns were identified: focal hyperecoic thickening of the intercavernosum septum (IS) with acoustic shadow (pattern 1), non-calcified thickening (isoechoic or slightly hyperechoic (pattern 2), and microcalcifications in the IS without associated acoustic shadow (pattern 3).

**Results:**

Patients’ mean age was 51.3±16.7. ED was the predominant disorder in 73.2% of patients, followed by penile pain and length loss in 19.5% and 7.3% of patients, respectively. 32(78.1%) patients showed the pattern 1, 6 (14.6%) pattern 2, and 3 (7.3%) pattern 3. Plaques size varied from 3 to 13 mm. The penile hemodynamic response to CDU reported abnormal findings distally to the septal plaques in 20 patients (<25cm/sec). Median left and right cavernosum artery flows measured a peak systolic velocity of 31cm/sec and 33 cm/sec, respectively.

**Conclusions:**

We believe that an US study with CDU provides a way to characterize, localize, and deliver treatment choice in patients with Peyronie's Disease.

## INTRODUCTION

Peyronie's Disease (PD) is defined as an acquired fibrosis within the tunica albuginea, usually causing deformity, pain and erectile dysfunction (ED) (1). The prevalence of PD in the general male population ranges between 3 and 9% (2). It is considerably higher than the prevalence reported in the past decade, because social stigma prevents many patients from seeking medical care. Therefore, non-palpable isolated septal plaques (ISP) without deformity of the penis are not usually diagnosed at physical examination (3). Primary care physicians and urologists have reported incorrect assumptions about the prevalence and natural history of PD which can negatively affect diagnosis and treatment. Imaging is often required to confirm the clinical diagnosis, value the extent of disease, and define the treatment. The role of ultrasound in the diagnosis of PD is well proved due to the high resolution gray-scale imaging, alone or in combination with colour-Doppler (4). Non-palpable ISP of the penis showed by ultrasound (US) study are likely present in a significant number of patients affected by ED and penile pain without deformity or curvature (3, 4). In this study, we wished to define the US patterns observed in patients investigated for ED or penile pain without curvature.

## MATERIALS AND METHODS

Between March 2008 and September 2016, we retrospectively reviewed the medical records of 386 patients who underwent an initial colour-Do-ppler ultrasonography (CDU) of the penis for DE and/or non-resolving penile pain without curvature. The criteria used to enrol patients were: inability to obtain or maintain sufficient penile erection < 1 year; an International Index of Erectile Function 5 score (IIEF5) < 21; penile pain without curvature or deformity < 6 months. For a standardization of the clinical data, patients with a history of traumatic penile injury, mental disorder, abnormal blood levels of sex hormones and history of penile deformity were excluded from our study. All patients were evaluated through their detailed history, whole blood counts, blood levels of sex hormones (testosterone, prolactin, luteinizing hormone and follicle-stimulating hormone) and IIEF5 questionnaires. During genital examination, the penis was inspected for presence/absence of plaques. Any penile deviation was observed in all patients enrolled. US study was performed with the patient in the supine position using a machine equipped with a 7-12 MHz multi-frequency linear probe. All patients provided written informed consent before the procedure. With the penis placed toward the abdomen and transducer placed at the ventral surface of the root of the penis, a first B-mode US study was performed in transversal and longitudinal planes starting at the level of the glans and moving down to the base of the penis. Using a 25 gauge insulin injector, 10 micrograms of prostaglandin E1 (PGE1) was injected into the left corpus cavernosum. After the intra-corporal injection, diameters of right and left cavernosum arteries were measured, and the peak systolic velocity (PSV) was estimated every 5 minutes for 25 minutes (5). Less than 10% of patients required additional doses. The values used for different vascular status definitions were PSV less than 25cm/sec for arterial insufficiency and end-diastolic velocity (EDV) greater than 5 cm/sec for corporal venous-occlusive dysfunction. CDU was performed by an experienced sonographer who has been performing CDU studies on patients with ED for more 15 years (LD). US evaluation of size, location, and morphological patterns of the plaques were recorded. Measurement of plaques length and width were made in the longitudinal and transverse axes to calculate the total area. In gray-scale ultrasonography, the two corpora cavernosa are homogeneous in echo structure and identified as two hypoechoic circular structures. The tunica albuginea is visualized as a linear hyperechoic structure covering the corpora cavernosa (6). The echoes from the tunica albuginea are specular reflections and thus are showed with efficacy only when the ultrasound beam is perpendicular to them. Calcified penile plaques are usually seen as focal hyperechoic thickening of the tunica albuginea, showing strong echogenicity with attenuation of the acoustic beam. However, non-calcified plaques are isoechoic or slightly hyperechoic compared with the surrounding tunica albuginea (3, 5). Peyronie's plaques are more often located on the dorsal side of the penis, but they can also be found ventrally or, less in other positions (7). A central plaque confined to intercavernosum septum (IS) could contribute to loss of rigidity distally to the lesion, penile length loss or pain, and changing of the blood flow without evidence of deformity (8).

### Statistical analysis

Descriptive statistics for variables with a normal distribution, non-normal distribution, and categorical variables were evaluated using mean and standard deviation or median and interquartile range, according to their distribution. The association between US patterns and the factors associated were evaluated using the Student's t-test or the Mann Whitney U test, depending or their distribution. Statistical analyses were performed using Microsoft Excel 2010 platform. A p<0.05 was considered to indicate statistical significance.

## RESULTS

**Figure 1:**
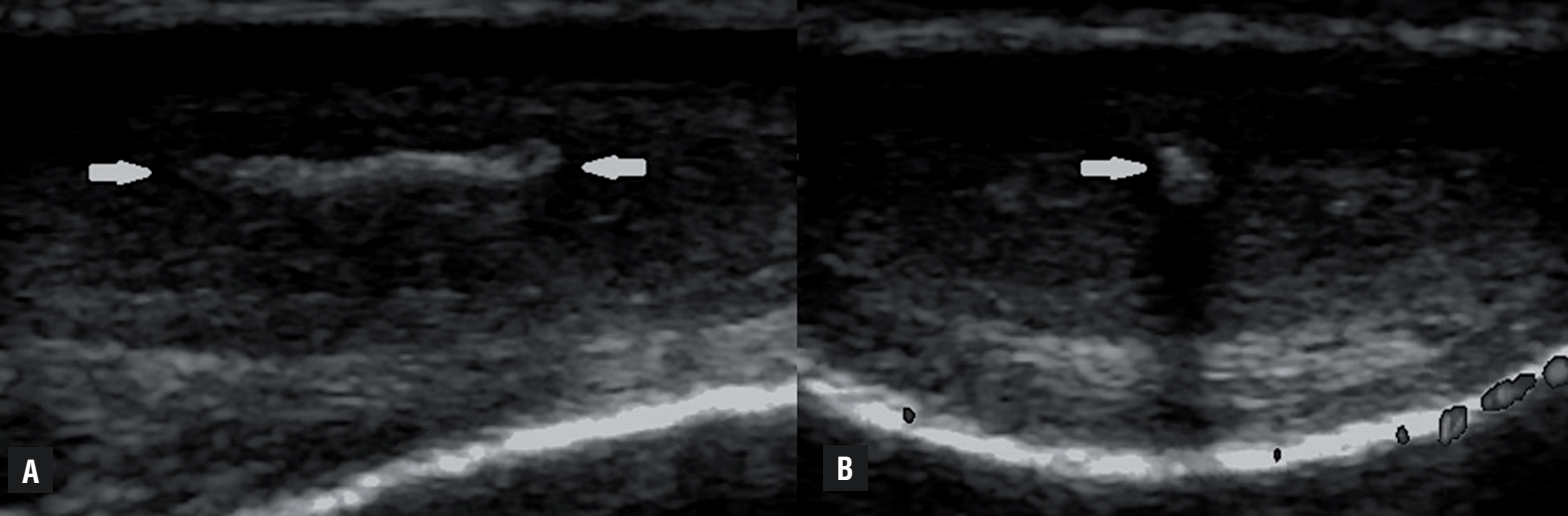
Longitudinal (A) and transverse (B) US images obtained along the ventral aspect of the penis show a hyperechoic thickening of the intercavernosus septum with acoustic shadow (white arrows).

**Figure 2:**
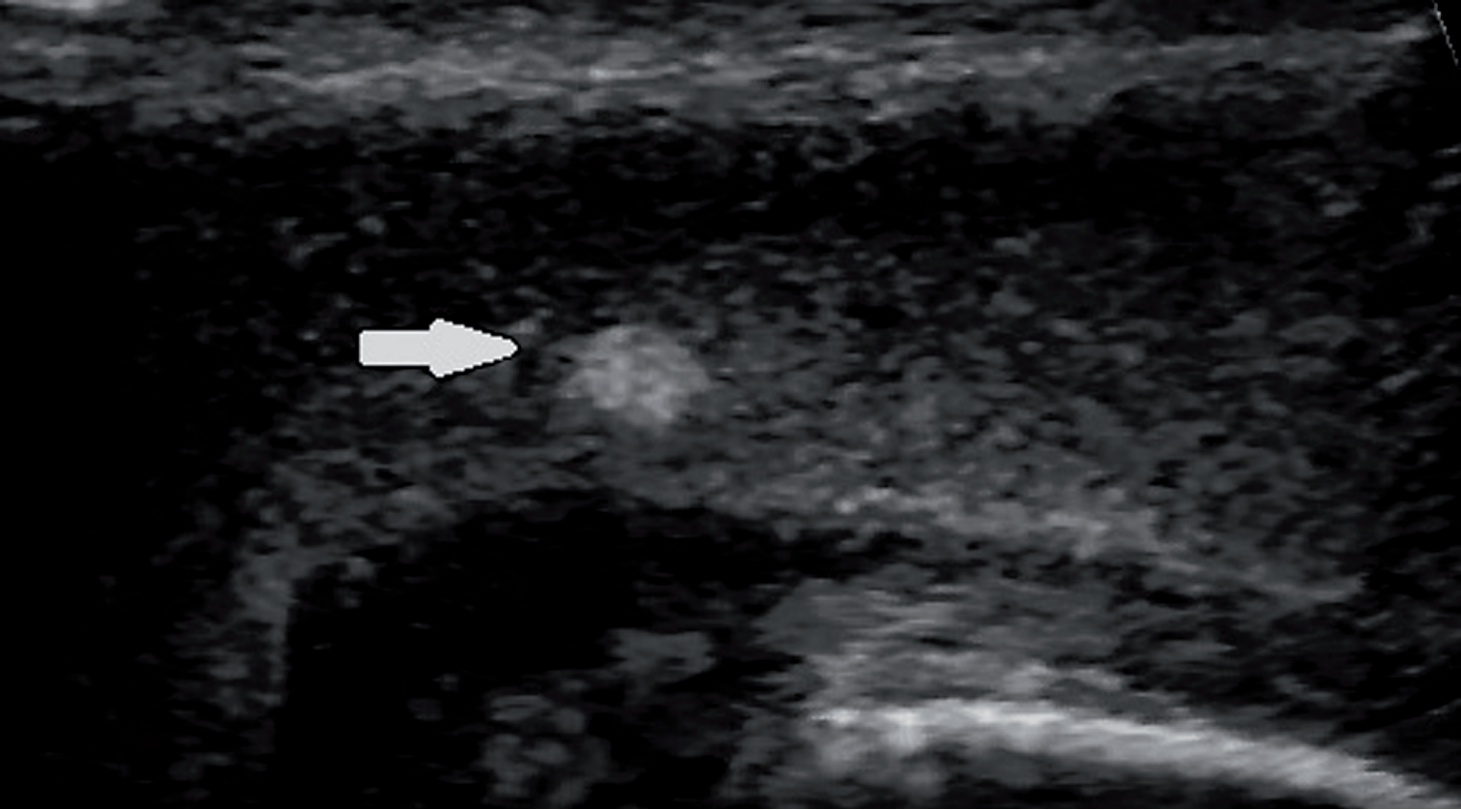
Longitudinal US image obtained along the ventral aspect of the penis shows a slightly hyperechoic thickening of the intercavernosus septum without acoustic shadow (white arrow).

**Figure 3:**
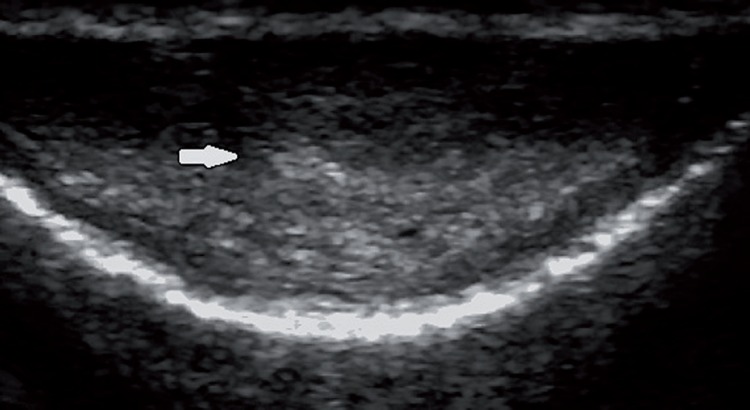
Transverse US image obtained along the ventral aspect of the penis shows microcalcifications of the intercavernosus septum without associated acoustic shadow (white arrow).

Of the 386 patients retrospectively analysed using our ultrasound database, we stored images of 41 patients that presented penile plaques confined only to the IS and complete clinical data. Among patients excluded from the study: 331 patients did not present plaques, 5 patients presented plaques also in other positions, and 9 patients presented plaques confined only to the IS, but their data were not complete, or images were not available for review. Patients’ age ranged from 29 to 68 years with mean age of 51.3±16.7. The baseline demographics and clinical characteristics of the 41 patients included in the study are shown in Table-1. The interval from onset of symptoms to clinical presentation was 6.8 (range 2-9) months. ED was the predominant disorder in 73.2% (30/41) of patients, followed by penile pain and length loss in 19.5% (8/41) and 7.3% (3/41) of patients, respectively. Only 12.2% (5/41) of patients reported a suspicious of penile trauma during sexual activity. Medical history revealed presence of risk factors for ED: hypertension (39%), cardiovascular diseases (17.1%), diabetes (9.8%), hypercholesterolemia (34.2%), and smoking history (39.1%). All patients had a non-palpable plaque and B-mode US allowed the recognition of plaque with involvement of the penile septum. Thirty two (78.1%) patients showed hyperechoic thickening of the IS with acoustic shadow (Figure-1), 6 (14.6%) patients non-calcified plaques (isoechoic or slightly hyperechoic compared with the surrounding tunica albuginea; Figure-2), and Figure-3 (7.3%) patients microcalcifications in the IS without associated acoustic shadow (Figure-3). Plaques size varied from 3 to 13 mm in maximum diameter. The penile hemodynamic response to intracavernosum PGE1 injection reported abnormal findings distally to the ISP in 20 patients (<25cm/sec). Median left and right cavernosum artery flows measured a PSV of 31cm/sec (range 17-80) and 33 cm/sec (range 15-80), respectively. The management of twenty-five patients included a conservative treatment with oral medication: phosphodiesterase-5 inhibitor (34.2%), vitamin E (22%) and pentoxifylline (4.9%). The other sixteen patients underwent intralesional injections with steroids (14.7%) and verapamil (24.4%). None of the patients has been subjected to surgical treatment.

**Table 1:** Distribution of clinical characteristics and ultrasonography patterns in patients affected by Peyronie's Disease with plaques confined to intercavernosus septum.

		US patterns			
Patients characteristics		Hyperechoic thickening with AS (n:32)	Isoechoic thickening (n:6)	Hyperechoic thickening without AS (n:3)	P
Age (years), median (range)		55.5 (45-63)	54.3 (29-68)	54.5 (48-61)	NS
Plaques size (mm), median (range)		7.9 (3-13)	8 (5-12)	7.6 (6-10)	NS
BMI (kg/m^2^), mean±SD		25.7±4.3	24.7±3.9	25.4±4.9	NS
**Tobacco, n (%)**					NS
	Never	19 (59.4)	4 (66.7)	2 (66.7)	
	Former	5(15.6)	0	1 (33.3)	
	Current	8 (25)	2(33.3)	0	
Hypertension, n (%)		12 (37.5)	3 (50)	1 (33.3)	<0.001
Cardiovascular diseases, n (%)		7 (21.9)	0	0	<0.001
Diabetes, n (%)		4 (12.5)	0	0	<0.001
Hypercholesterolemia, n (%)		9 (28.1)	3 (50)	2 (66.7)	<0.002
**Clinical symptoms, n (%)**					<0.001
	Erectile dysfunction	26 (81.3)	2 (33.3)	2 (66.7)	
	Penile pain	4 (12.5)	4 (66.7)	0	
	Penile length loss	2 (6.2)	0	1 (33.3)	
**Penile CDU findings, n (%)**					<0.001
	Mean PSV > 25 cm/sec	14 (43.8)	4 (66.7)	3 (100)	
	Mean PSV < 25 cm/sec	18 (56.2)	2 (33.3)	0	
	Mean EDV > 5 cm/sec	2 (6.2)	0	0	
	Mean EDV < 5 cm/sec	30 (93.8)	6 (100)	3 (100)	
**Treatments for PD, n (%)**					<0.001
	Phosphodiesterase-5 inhibitor	14 (43.8)	0	0	
	Vitamin E	2 (6.2)	4 (66.7)	3 (100)	
	Pentoxyfilline	2 (6.2)	0	0	
	I.I. with steroids	6 (18.8)	0	0	
	I.I. with verapamil	8 (25)	2 (33.3)	0	

**US** = ultrasonography; **AS** = acoustic shadow; **BMI** = body mass index; **NS** = not significant; **CDU** = color-Doppler ultrasound; **PD** = Peyronie's disease; **I.I** = intralesional injections.

## DISCUSSION

As a connective tissue disorder of penile tunica albuginea, PD commonly causes deformity and shortening of the penis often associated with ED (9). Moreover, it is the most frequent cause of penile pain (6). Although first observed in 1561 by Fallopius and Vesalius, it was not until 1743 that the disease was fully described by de la Peyronie (10). Over 270 years after the first description, the exact pathophysiology remains still uncertain (1-3). PD is thought to arise from microvascular trauma during sexual intercourse. In response to such trauma, inflammatory cells (macrophages, neutrophils, mast cells) release inflammatory mediators and collagenases. In this early phase of PD, inflammation and edema irritate nerve endings, thereby producing pain. Subsequently, in the chronic phase of PD, the process of plaque formation impairs the erectile tissue often resulting in ED, length loss and deformity of the penis (11, 12). The diagnosis of PD is based on: medical history, physical examination, photographic images as well as US imaging modalities with or without CDU, computed tomography (CT) and nuclear magnetic resonance (MRI) (13). Recently, a validated questionnaire was developed to help the diagnosis and evaluation of the gravity of PD, known as the Peyronie's Disease Questionnaire (PDQ). The PDQ is a 15-question tool to assess the presence, progression, and severity of symptoms in patients with PD (14). The role of imaging is to detect impalpable plaques and determine their dimensions. MRI is superior to US for superior contrast resolution in assessing non-calcified plaques. On MRI the plaques appear as hypo-intense areas of thickening in the tunica albuginea on both T1-weighted and T2-weighted sequences (5, 15, 16). Hauck E et al. (17) observed that MRI provides a detailed evaluation of penile anatomy, an accurate presentation of irregularities of tunica albuginea, as well as deformities of corporal bodies. On US, plaques calcification are better showed than on MRI with a detection rate of 100% (6). Calcifications are signal-free on MRI, but the use of gadolinium perifocal contrast enhancement can make inflammatory reactions in and around the calcified plaques (16). However, penile calcifications are not uncommon incidental finding on CT of the pelvis (18). Ultrasonography has been observed to be the method of choice because it is cost-effective and can define both morphological patterns and hemodynamic status of the corpora cavernosa (19). This study shows that non-palpable ISP of the penis identified by US are likely present in a significant number of patients who experience ED, pain or length loss of the penis without evidence of penile deformity or curvature. We evaluated the US patterns in attempt to identify the different stages of the disease and the lesions’ characteristics. Three distinct US patterns were observed, which correspond to different stages of PD. Penile plaques have usually been demonstrated as hyperechoic thickening of the IS with acoustic shadow (76.2%). Detection of plaque calcifications is associated with stabilization of the disease and provides information useful to select patients for lithotripsy therapy. Acoustic shadowing produced by extensive calcification of the plaques can reduce visibility of associated pathological changes of the corpora cavernosa (20). In accordance with several authors, we showed that isoechoic plaques are rare and characterized by focal thickening of the IS tissue (11). This form of presentation is found in the initial stages of the disease when the fibrosis is confined and the interstitial edema predominant (12). The isolated thickening and fibrosis of the septum represent the most challenging aspect of the disease, because it is more difficult to be identified on US study. The differential diagnosis includes the following conditions: cavernosum fibrosis secondary to local trauma, chronic inflammation, benign or malignant tumors (21). In particular, the epithelioid sarcoma of the penis is a rare disease that may express as focal lesion and can mimic PD (22). Finally, we described the US pattern of hyperchoic lesions in the IS without associated thickening and acoustic shadow. This pattern is reflective of a calcification process before solid plaque formation. However, microcalcifications can be occasionally identified also in regions in which the tunica albuginea is not thickened (6). Correlation between plaque enhancement characteristics and natural history of PD has been demonstrated by Bekos et al. (23). They observed that the density of echogenic areas and presence of acoustic shadows are predictors of disease's stability. Other authors don't confirm these results and to date, the natural history and mechanism of ISP remains undefined (8, 12). Bella et al. (24) reported that the IS acts as an inner supporting frame, resisting dorsal and ventral bending forces during tumescence. A trauma during intercourse causes pressure on the connection of IS, causing delamination of the septal fibers at the point of insertion. Devine et al. (25) showed that a repetitive trauma during intercourse might result in delamination between the layers of the IS and microvascular injury, which causes haemorrhage into the intralaminar space. The final results are production of fibroblasts and inflammatory mediators, and accumulation of collagen at the site of the injury. After plaque evaluation, a CDU study should be done, and erectile response of the patients should be evaluated. CDU of the cavernosum arteries gives information on penile blood flows, which is useful in planning medical or surgical treatments (15). Moreover, with the power mode, one can find hyper-perfusion around the plaques as a sign of inflammation in the active state of the disease (26). Lue et al. (27) reported a strong correlation between CDU results and clinical outcomes. The presence of IS fibrosis and tunical thickening were associated with decreased ability to have intercourse. As ED occurs in 30% in patients affected by PD (8, 12), however, penile vascular disease was common in our study (48.8%) because it was supported by the presence of cardiovascular risk factors in most of our patients.

## CONCLUSIONS

Multiple therapies have been offered for the management of PD, their efficacy is uncertain because numerous trials have lacked to demonstrate favourite findings. Because no single treatment is appropriate for everyone, it is critical to make an exact diagnosis prior to treatment, and factors such as plaque size, location, stability and ultrastructural alterations associated with the disease, should be determined prior to instituting any form of therapy. US has been shown to be a method of choice because it is cost-effective, painless, non-invasive, has no negative side effects and can define both ultrastructural patterns and corporal hemodynamic status. This US stratification may help therapeutic decisions making.

## ABBREVIATIONS

PD: = Peyronie's Disease
ED: = Erectile dysfunction
ISP: = Isolated septal plaques
US: = Ultrasound
CDU: = Colour-Doppler ultrasonography
IIEF5: = International Index of Erectile Function 5 score
PGE1: = Prostaglandin E1
PSV: = Peak systolic velocity
EDV: = End-diastolic velocity
IS: = Intercavernosum septum
CT: = Computed tomography
MRI: = Nuclear magnetic resonance
PDQ: = Peyronie's Disease Questionnaire
